# RoseNet: Rose leave dataset for the development of an automation system to recognize the diseases of rose

**DOI:** 10.1016/j.dib.2022.108497

**Published:** 2022-08-02

**Authors:** Sadia Sazzad, Aditya Rajbongshi, Rashiduzzaman Shakil, Bonna Akter, M. Shamim Kaiser

**Affiliations:** a Department of Computer Science and Engineering, National Institute of Textile Engineering and Research, Dhaka, Bangladesh; b Department of Computer Science and Engineering, Daffodil International University, Dhaka, Bangladesh; c Institute of Information Technology, Jahangirnagar University, Dhaka, Bangladesh

**Keywords:** Dataset, Rose leave, Feature ranking, Machine learning

## Abstract

For the welfare of self-development and the country's economic evolution, people invest their youth and money in different cultivation and sustainable production business sectors. The crops or fruits get all the attention for this purpose, but currently, the commercial cultivation of flowers is becoming a numerous beneficial investment. As a consequence, the rose(*Genus Rosa*) is one of the most beautiful and commercially demanding flowers among different flowers. However, insecticide resistance is considered one of the lion's share issues facing agricultural production of roses by decreasing plants' growth and the quality as well as the quantity of healthy-looking flowers. Apart from this, due to different natural and environmental issues, rose's quality and production level are losing their fame. Additionally, the cultivators of this sector are not educated enough to identify the initial affection of different diseases of leaves with beard eyes. Besides, the lack of communication skills to consult with an agriculturist timely turns the situation worst more than the estimation of the production. With this concern, early detection of diseases that affected different parts of roses, such as leaves, is crucial. Recently, image processing techniques and machine learning classifiers have been primarily applied to recognize multiple diseases. This article presents an extensive dataset of rose leaves images, both diseases affected and diseases free are classified into three classes (Blackspot, Downy Mildew, and Fresh Leaf). The dataset is composed of the collected images which were captured during the seasonal time of diseases affection with the consultation of a domain expert and the dataset is accessible at https://data.mendeley.com/datasets/7z67nyc57w/2.

## Specifications Table

**Table:** 

Subject	Computer Science
Specific subject area	Image processing, Image detection, Image classification, and computer vision.
Type of data	Images
How the data were acquired	First, the rose garden is chosen by examining the areas in Bangladesh where these gardens are most commonly found. Among numerous sites, an expert chose the rose garden, which has a large scale of rose production and a range of disease-affected leaves from December to February. Finally, using a semi DSLR camera, images were captured after visiting that location on the advice of an expert.
Data format	Raw
Description of data collection	The samples were not pre-treated in any way. With the help of a domain specialist and a plant research institute, the images were meticulously obtained from the rose garden.
Data source location	**Institution:** Village of roses (Golap Gram) **City:** Sadullapur Road Birulia Bridge, Dhaka 1216 **Country:** Bangladesh
Data accessibility	**Repository name:** Mendeley Data **Data identification number (permanent identifier, i.e., DOI number):**10.17632/7z67nyc57w.2 **Direct link to the dataset:**https://data.mendeley.com/datasets/7z67nyc57w/2

## Value of the Data

• As the cut rose industry grows, this dataset, when utilized in machine learning and deep learning models, aids in the early identification and categorization of rose leaf illnesses, which is critical for the commercial development of a very well computerized cultivation system.
• Researchers may utilize this data to create a digitalized system that investors may use to better mass production, reducing stress and contributing significantly to the global economy.
• Using multiple machine learning and deep learning models, the dataset of left illnesses may be used to segment, identify, and categorize early indicators of diseases detected in rose leaves. Because the data was obtained at the field level, the researchers will be able to produce relevant results using machine learning and deep learning models.
• Along with different machine learning methods to detect those diseases correctly, early detection is the most important for identifying diseases that can be assisted farmers to produce higher yields of products, resulting in improvement of their socioeconomic stability by overcoming the huge losses.
• Early detection of rose leave diseases is required for large-scale production; consequently, the dataset will assist the researcher and investors to overcome numerous challenges.

## 1 Data Description

Rose is a member of the Rosaceae family which are mostly native to Asia, North America, Europe, and northwest Africa. Since time immemorial, people have followed rose cultivation in different countries. Cut rose output, on the other hand, is declining and losing its quality and economic worth these days due to a number of illnesses that farmers were previously unable to detect with their naked eyes. The rose leaves are affected by numerous diseases such as black spots, rust, dieback, powdery mildew, etc. [1]. Because of those diseases, rose cultivation is hampered very badly. Among those illnesses, this article focuses on two particular diseases that affect rose plants and leaves. Furthermore, disease-free photos are included in machine learning and deep learning models for correct categorization. Table 1 has a thorough explanation of the dataset that was used.

**Table 1:** Dataset description.

Class Name	Symptoms	Visualization
Black Spot	Black spot is a common and dangerous rose disease that can spread quickly throughout a season. The fungus Diplocarpon rosae causes the black spot. It becomes worst in the spring when there's been a lot of rain, and it's been hot. On rose leaves, symptoms appear as round, black spots that expand to a diameter of 1/2 inches and are encircled by a yellow region. Infected leaves frequently fall off the plant. Throughout the summer, the illness persists [2].	

Downy Mildew	The viruses that cause downy mildew infect the leaves, but they can also infect the stems and fruits. They generate lesions on the top leaf surface, which are yellow at first, turn brown, and are surrounded by veins. Fluffy growth emerges on the underside of the leaf, which starts as white and then becomes gray-brown [3]. The leaf veins limit their growth as the lesions enlarge, giving the spots an angular appearance. As the lesions grow more prominent, more leaf damage occurs, and, eventually, the leaves drop off.	

Disease-free (leave)	The leaves are palmate intricate (beak) generally alternate, with oval, coarsely serrated leaflets. Wild rose flowers normally have five petals. In addition to being pinnate, Rose leaves also have a serrated margin. They range from 3 to 20 cm and sometimes have prickles on their underside [4].	

A total of 917 images were accomplished in this rose leave diseases dataset containing the disease affected and disease-free leaves images and the dataset is easily accessible at https://data.mendeley.com/datasets/7z67nyc57w/2. The images were collected from a prominent place in Dhaka, Bangladesh, called the village of roses (Golap Gram). This dataset is useful for agricultural researchers that use machine learning and deep learning models, particularly for rose leaves disease detection. With the support of professionals and researchers in this field, images of rose leaves are painstakingly captured by the semi DSLR Camera. After that, image processing techniques are used to enhance the assembled original image. The dataset acquisition process is described in Table 2. All captured images have been scaled to 512 × 512 pixels in size. The distribution of this is shown in Table 3.

**Table 2:** Data acquisition steps.

Serial. No	Acquisition time duration	Done activity
Image Collection	December to February	Rose flowers start blooming from the time of the month September to February. Thus, the peak time of getting the full bloomed flower was the middle of December to February. At that time, the images were captured with the help of natural and artificial light from various perspectives and backgrounds.

Image Augmentation	March	Researchers may need a verst amount of data to get more accuracy in deep learning model implementation. That's why the data was augmented. As a result, the total number of samples was raised from 917 to 4342.

**Table 3:** Distribution of dataset as class wise.

Name of Class	Amount of Original Data	Amount of Augmented Data
Black Spot	313	1434
Downy Mildew	200	1478
Disease-Free (leave)	404	1430
Total	917	4342

## 2 Experimental Design, Materials and Methods

### 2.1 Specification of Camera

The data was collected with a Sony Cyber-Shot H200 Semi DSLR Camera, which features a 20.1-megapixel picture sensor, a 26x optical zoom OIS lens, sophisticated flash, and iAuto Mode technology. Pixel Gross: 20.4MP; 1/2.3′′ Super HAD CCD Sensor; 3.0′′ Clear Photo LCD (460 K dots) with Brightness Control. Frame advance rate is set to 4fps manual focus, shutter speed is 1/250 s, and all other parameters are left alone.

### 2.2 Prepossessing

**Fig. 1:**
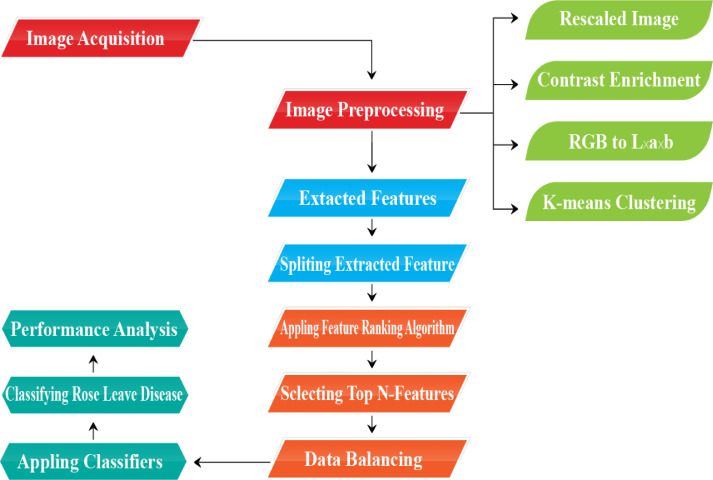
Generic working steps of the recognition of rose leave disease.

All the acquired images were gone through some steps such as (i) Image Acquisition (ii) Image Preprocessing (Rescaled Image, Contrast Enrichment, RGB to L*a*b, Segmentation) (iii) Extracted Features (iv) Splitting Extracted Features (v) Applying Feature Ranking Algorithm (vi) Selecting Top N-Features (vii) Data Balancing (viii) Applying Classifiers (ix) Classifying Rose Leave Diseases (x) Performance Analysis. As the images were collected by random clicking, the shape and size were not on a uniform scale. Thus, firstly the accumulated images needed preprocessing. As a result, the photos were downsized using the Bicubic interpolation method to a consistent size of 512,512 pixels. Along with this step named Rescaled Image, the other steps, one by one, named Contrast Enrichment to enhance the contrast of the image, RGB to L*a*b conversion by RGB color space which is feasible in L*a*b color space, and K-means Clustering used for image segmentation, were also performed as described in Fig. 1. Secondly, after completing those four sub-steps, the features were extracted from the preprocessed image. A detailed description of extracting features from the original image is depicted in Table 4.

**Table 4:** Manual procedure of feature extraction from the input image.

After that, the thirteen Gray Level Co-occurrence Matrix (GLCM) and Statistical features are removed [5]. Then the extracted features were split, and the feature ranking algorithm was applied. As a result, the top N-features were selected because all the extracted features were not performed well. The most ranked features were utilized for further preceding. Thirdly, the data balancing step was performed. Finally, those top-ranked balanced data were applied to train and test the adaptive classifier to evaluate the performance for classifying rose leave diseases. Our future goal is to establish an automated system to identify the diseases of rose and validate its performance.

## Ethical Approval (Involvement of Animals)

This article does not contain any studies with animals performed by any of the authors.

## Ethical Approval (Involvement of Human Subjects)

There are no studies involving human participants done by any of the authors in this article. The datasets used in the article are open to the public. For the usage of these datasets, proper citation rules should be maintained.

## CRediT authorship contribution statement

**Sadia Sazzad:** Conceptualization, Methodology. **Aditya Rajbongshi:** Data curation, Visualization, Writing – original draft. **Rashiduzzaman Shakil:** Software, Validation. **Bonna Akter:** Writing – review & editing. **M. Shamim Kaiser:** Supervision.

## Declaration of Competing Interest

The authors declare that they have no known competing financial interests or personal relationships that could have appeared to influence the work reported in this paper.

The authors declare the following financial interests/personal relationships which may be considered as potential competing interests:

## Data Availability

FlowerNet: An extensive rose leaves dataset for disease recognition applying machine learning and deep learning models (Original data) (Mendeley Data).
